# Impaired Function of CD5^+^CD19^+^CD1d^hi^ B10 Cells on IgE Secretion in an Atopic Dermatitis-Like Mouse Model

**DOI:** 10.1371/journal.pone.0132173

**Published:** 2015-08-05

**Authors:** Jieqiong Li, Chunping Shen, Ying Liu, Yunzhu Li, Lin Sun, Lei Jiao, Weiwei Jiao, Jing Xiao, Chen Shen, Hui Qi, Fang Xu, Lin Ma

**Affiliations:** 1 Key Laboratory of Major Diseases in Children Ministry of Education, National Key Discipline of Pediatrics (Capital Medical University), Beijing Key Laboratory of Pediatric Respiratory Infection Diseases, Beijing Pediatric Research Institute, Beijing Children’s Hospital, Capital Medical University, Beijing, 100045, China; 2 The Department of Dermatology, Beijing Children’s Hospital, Capital Medical University, Beijing, 100045, China; University Hospital Hamburg-Eppendorf, GERMANY

## Abstract

Atopic dermatitis (AD) is a chronic inflammatory pruritic skin disease in which the pathogenic mechanism is complicated and not completely understood. Reports on the role of regulated cells in AD have recently evolved to regulate B cells, which may play a role in allergic inflammation as well. In the present study, we examined the frequency and regulatory function of CD5^+^CD19^+^CD1d^hi^ B10 cells in an AD-like mouse model. Our results showed that the percentage of CD5^+^CD19^+^CD1d^hi^ B10 cells increased while the frequency of IL-10-producing B cells in CD19^+^B cells decreased in the mice of AD group. Moreover, no difference in the percentage of B10pro+B10 cells was observed between the AD and control groups. Strikingly, B10 cells from control mice effectively inhibited IgE secretion, whereas the suppressive function of B10 cells from the AD mice was significantly decreased, which was similar to that observed in the group without B10. Altogether, these results suggest that the number of IL-10-producing B cells decreased in the AD group and these cells showed a defective regulatory function on IgE secretion.

## Introduction

Atopic dermatitis (AD), a complex skin disease consisting in two phases (acute and chronic), is generally characterized by an increase in serum IgE levels [1]. This disease is a systemic disorder caused by skin barrier dysfunction, severe skin dehydration, and mutations in the filaggrin gene, which plays an essential role in modulating epidermal homeostasis [2]. Previous studies have shown that the developmen to fatopic skin lesions is associated with an imbalanceof T cells such as Th1/Th2, Th17/Th22 and regulated T cells (Treg) in the immune system [3].

Interleukin 10 (IL-10) is a potent anti-inflammatory cytokine that has been associated with AD. Compared to mild AD and normal controls, a higher number of IL-10-producing CD4^+^ T cells were observed in severe AD patients [4]. In the majority of patients, plasma levels of IL-10 also seem to reciprocally correlate with the severity of AD [5]. Furthermore, the importance of IL-10 in the maintenance of AD has been recently highlighted by the idea that Toll-like receptor 2 ligands promote chronic AD through IL-10 [6]. Although the main cellular source of IL-10 is Treg cells [7], the regulatory B cells, which are a subset of B cells, have recently emerged as another important resource for IL-10 [8–10].

In general, B lymphocytes express different cell-surface immunoglobulin receptors that recognize specific antigenic epitopes [11]. Activated B cells are generally considered to play a critical role in AD by stimulating CD4^+^T-cell proliferation and Th2/Th17 responses [12]. However, recent studies have described anew subset of B cells, regulatory B cells (Bregs),which are capable of downregulating immune responses in mice and humans [8–10, 13].

Two majorBregpopulations have been reported in mice. The precursor Breg cells of the marginal zone (T2-MZP B cells) in a collagen-induced arthritis (CIA) mouse model were described by Evans et al. [14]. Subsequently, Tedder and colleagues described another subset of Breg cells that expressed the CD5^+^CD19^+^CD1d^hi^phenotype. This B-cell subset was called B10 cells andwas believed to account for most of the IL-10 secreted by B cells in mice [8]. B10 cells (1–2%) are detectedmainlyin the spleen and are defined by its unique capacity to produce IL-10after 5 h of *in vitro* stimulation [8]. Furthermore, B10 progenitor (B10pro) cells,which can be induced to mature by stimulation using agonistic CD40 monoclonal antibody (mAb) for 48 h, havealsobeen identified within the spleen CD5^+^CD19^+^CD1d^hi^ B-cell subset [8]. Indeed, the regulated functions of B10 cells are strictly related to their capacity to produce IL-10 [9, 15–18] and have been investigated in mouse models of autoimmune diseases such as contact hypersensitivity [8], lupus [19, 20], experimental autoimmune encephalomyelitis (EAE) [21–23], inflammatory bowel disease (IBD) [24, 25], and graft-versus-host disease [26]. In addition, there is growing evidence that IL-10-producing B cells also play animportant role in human autoimmune diseases such as lupus and rheumatoid arthritis [27, 28]. However, the role of B10 in the pathogenesis of AD has not been fully elucidated.

The findings of previous studies have prompted us to determine whether B10 cells also play a suppressive role in allergic inflammation that is associated with AD. Therefore, using a well established mouse model, we investigated the participation of B10 cells, the main type of Breg cells, in the mechanism underlying their protective properties.

## Materials and Methods

### Animals

Six-week-old female BALB/c mice were purchased from the Academy of Military Medical Sciences (Beijing, China). All mice were maintained in specific pathogen-free environments and standard diet and water were provided by the lab. All animal experiments were approved by the Institutional Animal Care and the animal ethics committees of Capital Medical University [permit number: SCXK (JING2012-0001)].

### Induction of dermatitis and evaluation of skin lesions

The BALB/c mice were divided into two groups, namely the AD group (n = 10) and the control group (n = 10). DNFB (Wako, Japan) diluted in the mixture of acetone and olive oil (4:1) was used for sensitization [29]. The dorsal side of the mice was shaved on the first week of the experiment. Subsequently, the solution DNFB (100μL of 0.5% DNFB) was applied onto the backs of the mice in the AD group for sensitization. Then, after 4 weeks, 100μL of 0.2% DNFB was applied twice a week to develop lesions. Compared to the AD group, PBS was used in the control group and was applied for the entire procedure.

AD-like symptoms were evaluated by the development of dryness, erosion, excoriation, and hemorrhaging of the skin. The symptoms were scored as 0 (none), 1 (mild), 2 (moderate), and 3 (severe). Evaluation was performed by two volunteers and averages were calculated.

### Histological evaluation and measurement of epidermal thickness

Before the mice were sacrificed, the skin lesions were photographed, and sections were stained with hematoxylin and eosin (H&E). CD4^+^ T cells and mast cells were quantified by counting 8–10 fields (HPV at a magnification of 6,200×) per mouse in each group (n = 10). Each mouse was observed under the same microscopic magnification of 100× (Nikon E600).

The thickness of the dorsal skin and the length of the spleen were measured by using a micrometer (Hautine International Co, China) before and after the mice were sacrificed, respectively (n = 10). Three different sites of the back skin were measured by two volunteers and averages were calculated [29].

### Flow cytometric analysis

For flow cytometric analysis, splenocytes were isolated from each group (n = 10) using a 40-mm nylon cell strainer (BD Biosciences, San Jose, CA, USA). The cells were stained with the following monoclonal antibodies: fluorescein isothiocyanate (FITC) PE-Cy7-conjugated anti-CD5 (BD Biosciences), (FITC)-conjugated anti-CD19 (BD Biosciences), and phycoerythrin (PE)-conjugated anti-CD1d (clone 1B1; BD Biosciences) [8].

To detect the IL-10-producing B cells, the splenocytes of mice from each group (n = 10) were isolated according to the manufacturer’s instructions [30], and then cultured with LPS (10 μg/mL; Sigma Aldrich Chemie GmbH, Munich, Germany), phorbol 12-myristate 13-acetate (PMA; 50 ng/mL), ionomycin (iono; 500 ng/mL), and monensin (2 mM) for 5 h. For IL-10 detection, Fc receptors were blocked with mouse Fc receptor-specific mAb (BD PharMingen) prior to cell-surface staining and then fixed and permeabilized with a Cytofix/Cytoperm kit (BD PharMingen) according to the manufacturer’s instructions.

B10pro cells were defined as those B cells that are not induced to express cytoplasmic IL-10 after stimulation by using LPS + PMA + iono + monensin (L+PIM) for 5 h, but can be induced to mature into IL-10 competent B10 cells by *in vitro* culture with agonistic CD40 monoclonal antibody (mAb) stimulation for 48 h [8]. For detection of mouse regulatory B10pro cells, the splenocytes of mice from each group (n = 10) were isolated according to the manufacturer’s instructions, and CD40 monoclonal antibody (mAb) was added to induce B10 cells to mature. During the last 5 h of culturing, L+PIMwere added. Regulatory B10pro+B10 cells were magnetically sorted.

### Extraction of total RNA and real-time PCR

RNA of splenocytes was isolated using the TRI reagent (Molecular Research Center) as previously described [31]. Reverse transcription was performed using a First-Strand cDNA Synthesis kit (Promega). Real-time quantitative PCR was performed with TaqMan Universal Master Mix II or Brilliant III Ultra-Fast SYBR Green QPCR Master Mix (Agilent Technologies) with a StepOnePlus Real-Time PCR (Applied Biosystems). Real-time PCR results were analyzed using StepOne software that measured the amplification of the target and the endogenous control in samples and in a reference sample. The data were normalized to an endogenous control.

### Enzyme-linked immunosorbent assay (ELISA)

Mouse serum samples that were collected after the final sensitization were analyzed for IgE levels by using ELISA kits (Dakewei Bio, China).

Three days after the final sensitization, the splenocytes of mice from each group (n = 5) were isolated, and the CD5^+^CD19^+^CD1d^hi^ B10 cells were sorted from the spleen cells. In an *in vitro* culture, peripheral blood mononuclear cells (PBMCs) with CD5^+^CD19^+^CD1d^hi^ B10 cells were collected from each group and grown in 48-well U-bottom plates (250 μL/well) at 37°C for 3 days. During the last 5 h, LPS (10 μg/mL), PMA (50 ng/mL), and iono (500 ng/mL) were added (without monensin). Subsequently, the supernatant was collected for IgE level analysis.

### Statistical analysis

Aggregate data are presented as means ± SEM. For non-parametric data, the Mann- Whitney U-test was used to analyze individual groups. All analyses were performed by using the GraphPad Prism software (GraphPad Software Inc, San Diego, CA). A P value of < 0.05 was considered significant in all analyses.

## Results

### The frequency of CD5^+^CD19^+^CD1d^hi^ B10 cells increased as the IL-10 levels decreased in the AD group

To induce AD-like skin lesions in mice, DNFB was repeatedly painted onto the backs of each mouse. Control to normal mouse (Figure A in S1 File), potent inflammatory changes, such as edema, desquamation, epidermal hyperplasia, as well as a high number of inflammatory cells infiltrating into the dermis were observed in AD mouse during the histopathological assessment of the skin lesions (Figure B in S1 File). Skin-infiltrating CD4^+^T cells and mast cells are generally a characteristic feature of AD. In our experiments, the number of CD4^+^T cells and mast cells was markedly higher in DNFB-exposed skin sites compared to that observed in the controls (Figure C in S1 File, P < 0.01). Furthermore, in the AD group, an increase in skin thickness was observed aftertwo weeks stimulation and this continued to increase until the mice were sacrificed. On the other hand, no difference was observed in BALB/mice treated with PBS until the last day of the experiment (Figure C in S1 File, P < 0.05). In accordance with the observed increase in skin thickness, the score of skin lesions (Figure C in S1 File, P < 0.01) and the length of the spleen (Figure C in S1 File, P < 0.01) were also higher in the AD group. Moreover, the IgE levels of the mice in AD group were also significantly higher than that observed in the controls (Figure C in S1 File, P < 0.01).

**Fig 1:**
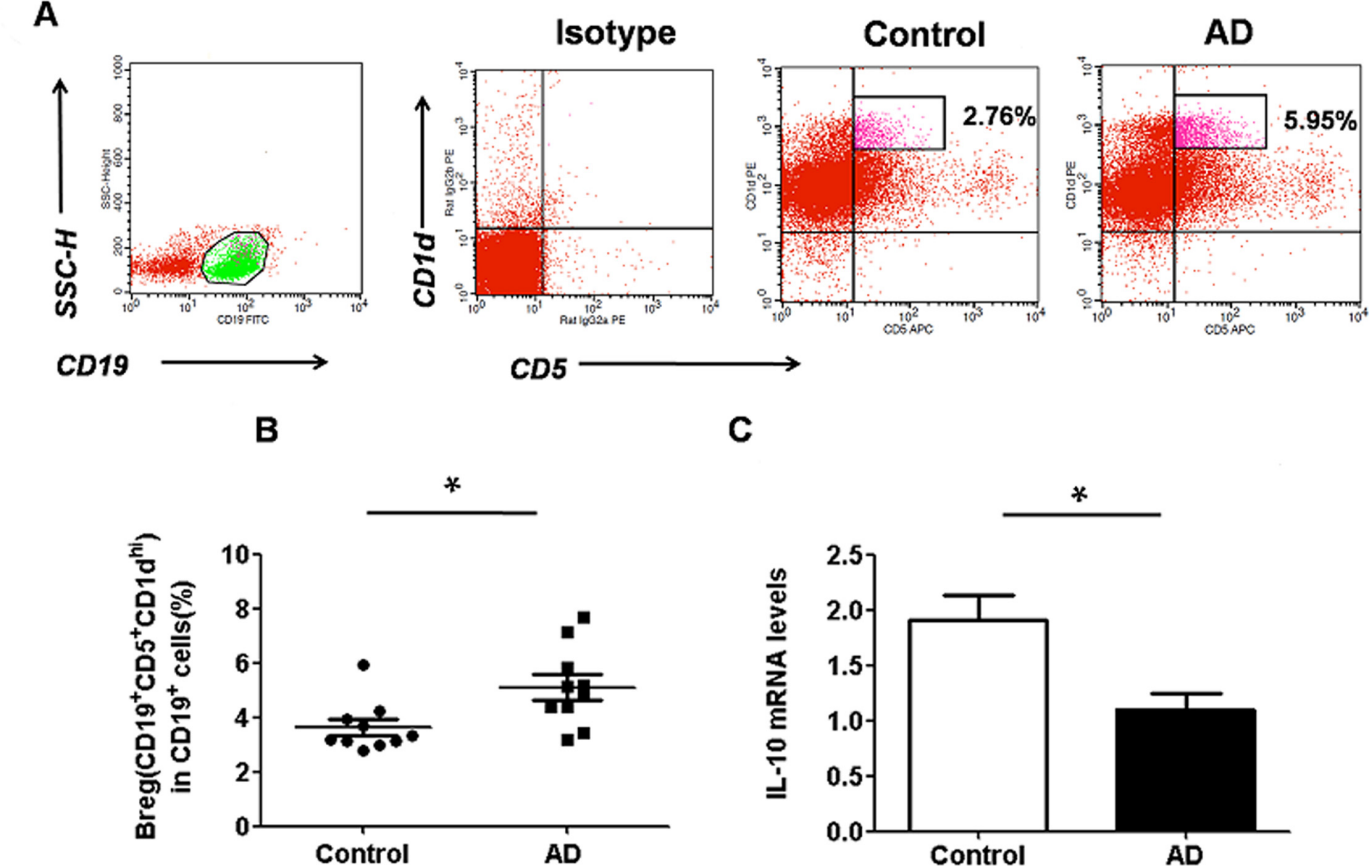
Regulatory B10 cells are augmented and the level of IL-10 expression is decreased in the spleen of AD mice. (A) Representative dot-plots showing the percentage of CD5^+^CD19^+^CD1d^hi^ B10 cells in control (n = 10) and AD mice (n = 10); (B) Bar graph depicting the percentage of CD5^+^CD19^+^CD1d^hi^B10 cells in control and AD mice as analyzed by flow cytometry. The percentage of CD5^+^CD19^+^CD1d^hi^ B10 cells was significantly higher in the AD group than that observed in the control. Data are expressed as mean ± SEM.*P<0.05, asanalyzed by one-way ANOVA, followed by Tukey multiple-comparison test; (B) The level ofIL-10 mRNA expression decreased in the spleen of AD mice (n = 10) compared to that of control (n = 10). Data are expressed as mean ± SEM. *P < 0.05, as analyzed by one-way ANOVA, followed by Tukey multiple-comparison test.

To determine whether regulatory B10 cells are relevant to AD, the well-described model of AD was used in the present study. DNFB was repeatedly painted onto the backs of each mouse. Two days after the last sensitization, the frequency of CD5^+^CD19^+^CD1d^hi^ B10 cells in the spleen was analyzed. As shown in Fig 1A and 1B, the percentage of CD5^+^CD19^+^CD1d^hi^ B10 cells was significantly higher in the AD group compared to that observed in the controls (P < 0.05).

To further validate the findings of flow cytometric analysis, the IL-10 levels of the spleen were also determined (n = 10). Surprisingly, different from that observed in the percentage of CD5^+^CD19^+^CD1d^hi^ B10 cells (Fig 1B, P < 0.05), the spleen IL-10 level of the AD group was lower than that observed in the controls (Fig 1C, P < 0.05). Taken together, these data indicated that the frequency ofCD5^+^CD19^+^CD1d^hi^ B10 cells increased while the IL-10 levels decreased in the mice of AD group.

### The capability of CD19^+^B cells to produce IL-10 was altered in AD group

**Fig 2:**
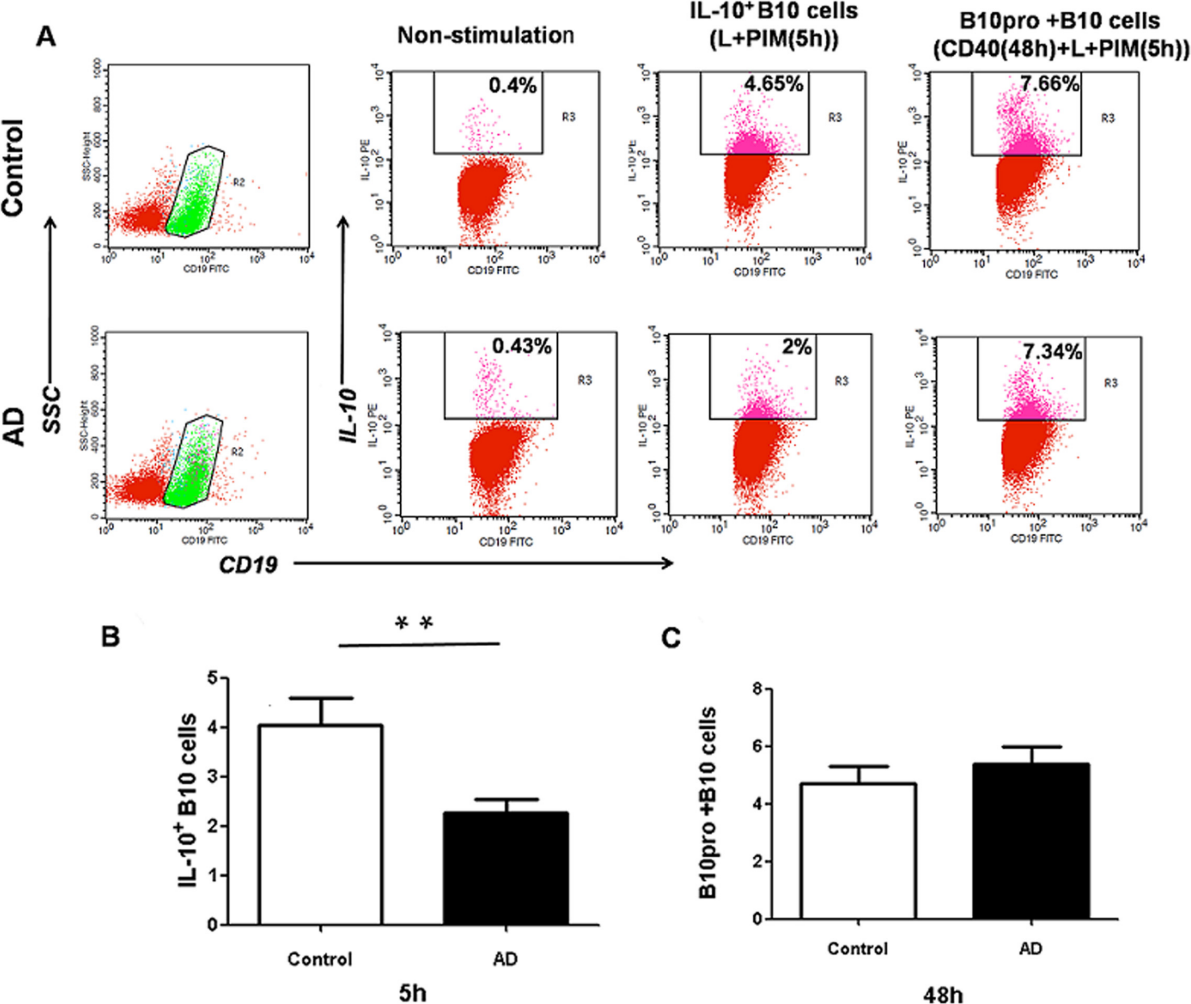
Comparison of IL-10 production in CD19^+^ B cells of AD mice and control. The B10 cells in the spleen of control (n = 10) or AD mice (n = 10) were *in vitro* stimulated for 5h or 48h withLPS(10μg/mL),PMA (50ng/mL), iono(500 ng/mL),monensin (2 mM), and CD40(1μg/mL). The number of CD19^+^IL-10^+^B cells wasmeasured by flow cytometry by using intracellular cytokine staining. (A) Representative comparative phenotype of CD19^+^IL-10^+^B cells from a control and AD mouse at 5 h and 48 h of stimulation; (B) The mean (±SEM) frequency of CD19^+^ B cells in PBMCs of spleen between controland AD groups after cultured for 5 h; (C) The mean (± SEM) frequency of B10pro+B10 cells in PBMCs of spleen between control and AD groups after culturing for 48 h; Data are expressed as mean ± SEM. Data are expressed as mean ± SEM. **P < 0.01, as analyzed by one-way ANOVA, followed by Tukey multiple-comparison test.

To determine whether IL-10 production in CD5^+^CD19^+^CD1d^hi^ B10 cells remained intact, spleen cells from AD or control mice were cultured with L+PIM [21]. L+PIM-stimulation for 5 h could induce IL-10 production of B10 cells. As shown in Fig 2, intracellular staining for IL-10 was observed after stimulation (Fig 2A). Although the percentage of IL-10-producing B cells in both groups significantly increased upon stimulation (data not shown), the percentage of IL-10-producing B cells in the AD group was lower than that observed in the controls (Fig 2B, P < 0.01). Combining the frequencies of CD5^+^CD19^+^CD1d^hi^B10 cells (Fig 1B), our results indicated that B10 cells from AD likely had an intrinsic defect that affected IL-10 expression after term stimulation.

B cell development is regulated through CD40; therefore the addition of CD40 mAb to stimulated cultures detects B10pro+B10 development [30]. To detect changes in B10pro+B10 cells, CD40 was added 43 h before L+PIM stimulation. Strikingly, no difference in the percentage of B10pro+B10 cells was observed between the AD and control groups (Fig 2C, P > 0.05). In conclusion, IL-10-producing B cells in AD mice had a lower capacity of producing IL-10 after 5 h of stimulation than that observed in the controls, whereas no difference in the frequency of B10pro+B10 cells was detected between these two groups, indicating the dysmaturity of B10pro cells in the spleens of AD mice.

### IgE levels of PBMCs cultured with B10 cells was altered

**Fig 3:**
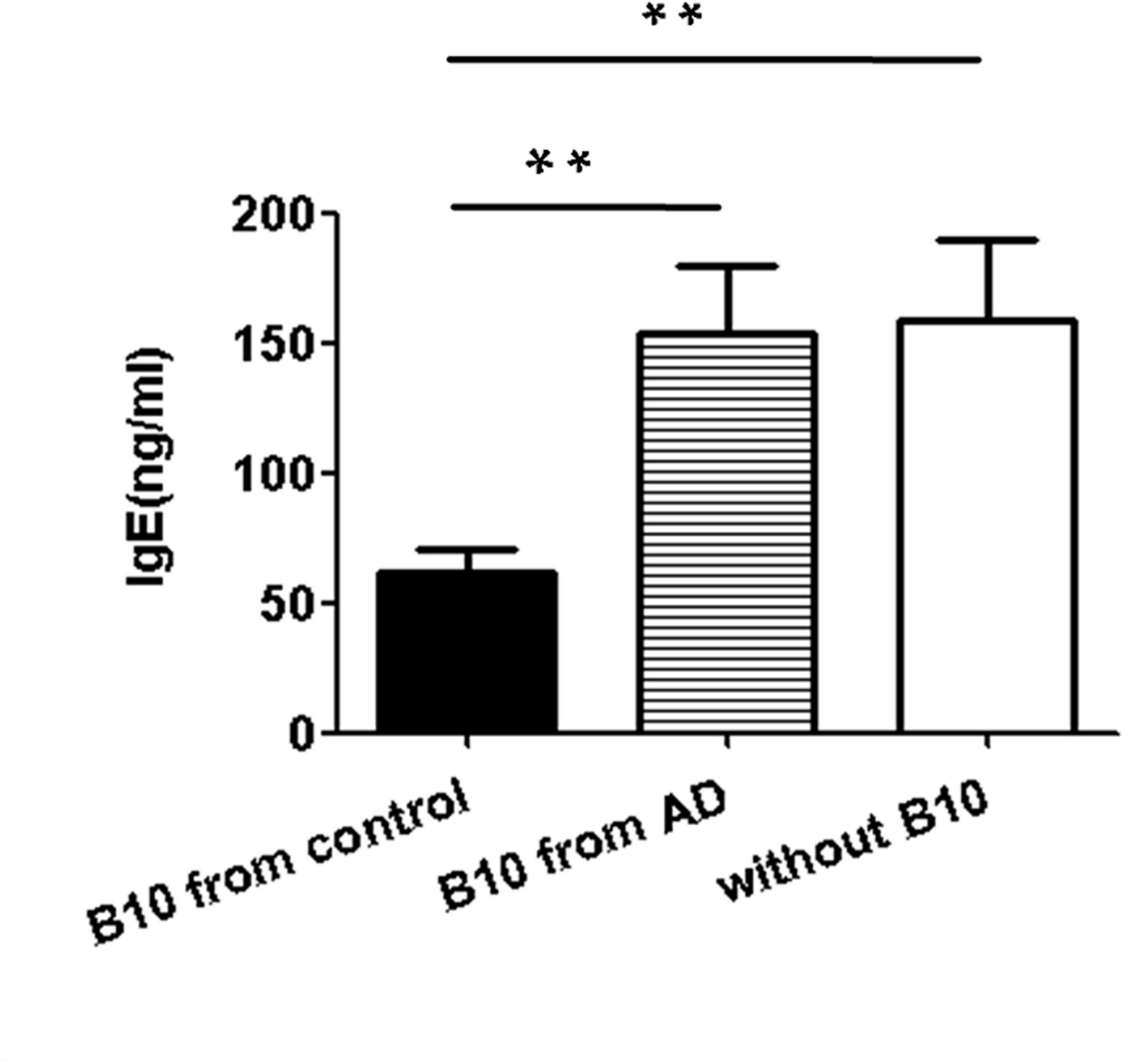
Influence of CD5^+^CD19^+^CD1d^hi^ B10 cells on IgE production. B10 cells were sorted from the spleen of AD mice (n = 5) and control mice (n = 5) by flow cytometry. The PBMCs from normal mice and B10 cells from control or AD mice were cultured with LPS (10 μg/mL), PMA (50 ng/mL), and iono (500 ng/mL) for 72h,then the IgElevels were determined in the supernatants by ELISA.PBMCs without B10 cell wereused as a negative control. No differences in IgE levels were observed in the AD and negative control groups, and both groups showed significantly higher IgElevel than that observed in the control group. Data are expressed as mean ± SEM. **P < 0.01, as analyzed by one-way ANOVA, followed by Tukey multiple-comparison test.

AD is a chronic inflammatory skin disease that is characterized by an increase in serum IgE levels. To further identify the regulatory function of B10 cells in IgE antibody-producing cells, we sorted B10 cells from the spleen of mice. These experiments were performed on PBMCs. Purified B10 cells sorted from AD or control mice were cultured with PBMCs for 72 h and LPS+PMA+iono were added at the last 5 h. At the same time, PBMCs without B10 cells were used as negative control. No differences in IgE levels between PBMCs without B10 cells and PBMCs cultured with AD B10 cells was observed (Fig 3, P > 0.05). However, the IgE level of PBMCs cultured with control B10 cells was significantly lower than that observed in the other two groups (Fig 3, P < 0.01). These results indicated that B10 cells from control mice were capable of efficiently suppressing IgE production by PBMCs whereas dysfunctional of B10 cells from AD group on IgE secretion was observed.

## Discussion

AD is a complex allergic condition that is characterized by high IgE levels, and IgE-mediated mechanisms are thought to play an important role in the pathogenesis of this disease [1]. In the present study, we applied DNFB as a hapten to BALB/c mice for 5 weeks to induce dermatitis, with an immune profile of higher levels of IgE, which was similar to the early reaction of AD [29].

In this report, we introduce regulatory B10 cells as important players in the pathogenesis of the IgE-mediated form of AD. By using a mouse model, we confirmed the dysfunction of B10 cells, which showed failure in suppressing IgE production in AD. The present work highlights a novel pathway by which regulatory B10 cells contribute to AD pathogenesis.

Recently, B cells have emerged as pleiotropic cells with diverse functions beyond antigen presentation and antibody production [9, 10]. B cells have been considered as modulators of the immune response because of their capacity to secrete cytokines. In contrast to human Breg cells, the surface markers of mouse Bregcellshave been defined as CD5^+^CD19^+^CD1d^hi^ B10 cells [8]. Here, we demonstrated that the number of splenic CD5^+^CD19^+^CD1d^hi^B10 cells in AD mice was significantly higher than that observed in normal controls. To date, Breg cells have not been well studied in AD, except for two recent investigations conducted by Geunwoong Noh et al. [32, 33]. Their researches demonstrated that the number of Breg cells decreased in the milk allergy group compared to that observed in the milk-tolerant group in AD patients, indicating the regulated function of Breg cells in the immune tolerance by producing IL-10. Our data are also consistent with the findings of previous studies on autoimmune diseases such as autoimmune encephalomyelitis and EAE [21–23].

In addition to cellular markers, regulatory B10 cells are defined by their ability to secrete IL-10 upon activation. This critical role of B cell-derived IL-10 was first demonstrated in a murine model of chronic colitis, in which the absence of Breg cells has been identified as the probable cause of the disease [34]. After that, the regulatory role of IL-10 secreted by B cells was confirmed in several human autoimmune diseases such as lupus [19, 20], EAE [21–23], and IBD [24, 25]. To characterize the participation of regulatory B10 cells in the process of AD, we analyzed the percentage of IL-10-producing B cells and the splenic IL-10 levels. Our results showed that the percentage of IL-10-producing B cells and the IL-10 levels in AD mice were both lower than that observed in the control group, which suggested that B10 cells from AD might have an intrinsic defect that affected IL-10 expression after term stimulation.

B10pro cells have also been functionally identified within the splenic cell subpopulation in mice. The B10pro cell subpopulations may acquire the ability to similarly function as B10 cells after 48 h *in vitro* stimulation by CD40 [8]. The present study shows that after 48 h of stimulation, no functional differences in B10pro+B10 cells between the AD and control groups were observed. Compared to 5 h of stimulation, the frequency of CD19^+^IL-10^+^B cells in the AD group was significantly lower than that observed in the controls. These results indicated the maturation arrest of B10pro cells in the AD group.

We further investigated the regulatory function of B10 cells because their suppressive activity might have influenced the secretion of IgE. Indeed, we found that PBMCs cultured with B10 cells, when isolated from AD mice, failed to suppress IgE production. However, B10 cells from control mice were capable of efficiently suppressing IgE production by PBMCs. The results of *in vitro* assays demonstrated that B10 cells from AD mice showed an impaired function of downregulate IgE production in PBMCs. Previously studies have shown that there were no differences in IgG/IgM expressing in PBMCs when these were cultured with Breg cells from pemphigus patients or healthy controls. Their failed results might attributable to the extremely low number of cells in the culture system, which were largely below the level of detection. It is also possible that B10 cells might mediate CD4^+^T cells, thus suggesting an autoimmune disorder associated with a Th2 response [35].

On the other hand, AD is an autoimmune disorder associated with the imbalance of CD4^+^T cells during immune responses such as Th1/Th2 bias [3]. Previous studies have shown that B10 cells could influence disease progression by stimulating Tcell proliferation and differentiation. They further showed that IL-10 from B10 cells also inhibited the differentiation of Th1 cells and this might contribute to the Th1/Th2 bias [36]. Taken together, B10 in AD mice might be functionally capable of suppressing CD4^+^ T cells differentiation and this will be the focus of our further research.

B cells play both positive effector and negative regulatory roles in the immunological system. Previous studies have shown that effector B cells contribute to the improvement of AD [12]. The present study has shown that the splenic B10 cell subset plays a critical role in the pathogenesis of AD in mice. The findings of the present study suggest the importance of maintaining the balance between opposing positive and negative regulatory functions changes in the pathogenesis in AD.

## Conclusions

In summary, we examined the frequency and regulatory function of CD5^+^CD19^+^CD1d^hi^ B10 cells in an AD mouse model. Our results showed that the percentage of CD5^+^CD19^+^CD1d^hi^ B10 cells increased while the frequency of IL-10-producing B cells in CD19^+^B cells decreased in the mice of AD group. Moreover, no difference in the percentage of B10pro+B10 cells was observed between the AD and control groups. Strikingly, B10 cells from control mice effectively inhibited IgE secretion, whereas the suppressive function of B10 cells from the AD mice was significantly decreased, which was similar to that observed in the group without B10. Altogether, these results suggest that the number of IL-10-producing B cells decreased in the AD group and these cells showed a defective regulatory function of IgE secretion.

## Supporting Information

S1 File Comparison of AD-like skin lesions in BALB/c mice after stimulation. (Figure A) Skin lesionsand histopathological images of corresponding skin of control mouse; (Figure B) Skin lesionsand histopathological images of corresponding skinofAD mouse. Significant erythema, desquamation, and crusting could be seen on the dorsal skin of model group; (Figure C) Comparison ofthe number of CD4^+^ T cells (per 100× field), the number of mast cells(per 100 × field), the thickness of skin, the score of skin lesions, the length of the spleen, and the IgE levels between control (n = 10) and AD (n = 10) groups. Data are expressed as mean ± SEM. *P < 0.05, **P < 0.01, AD *vs*.control.
(TIF)
