# Supplementary figures and images for: Impaired Function of CD5+CD19+CD1dhi B10 Cells on IgE Secretion in an Atopic Dermatitis-Like Mouse Model
Source: PLoS One. 2015 Aug 5;10(8):e0132173. doi: 10.1371/journal.pone.0132173 (PMC4526574; doi:10.1371/journal.pone.0132173)

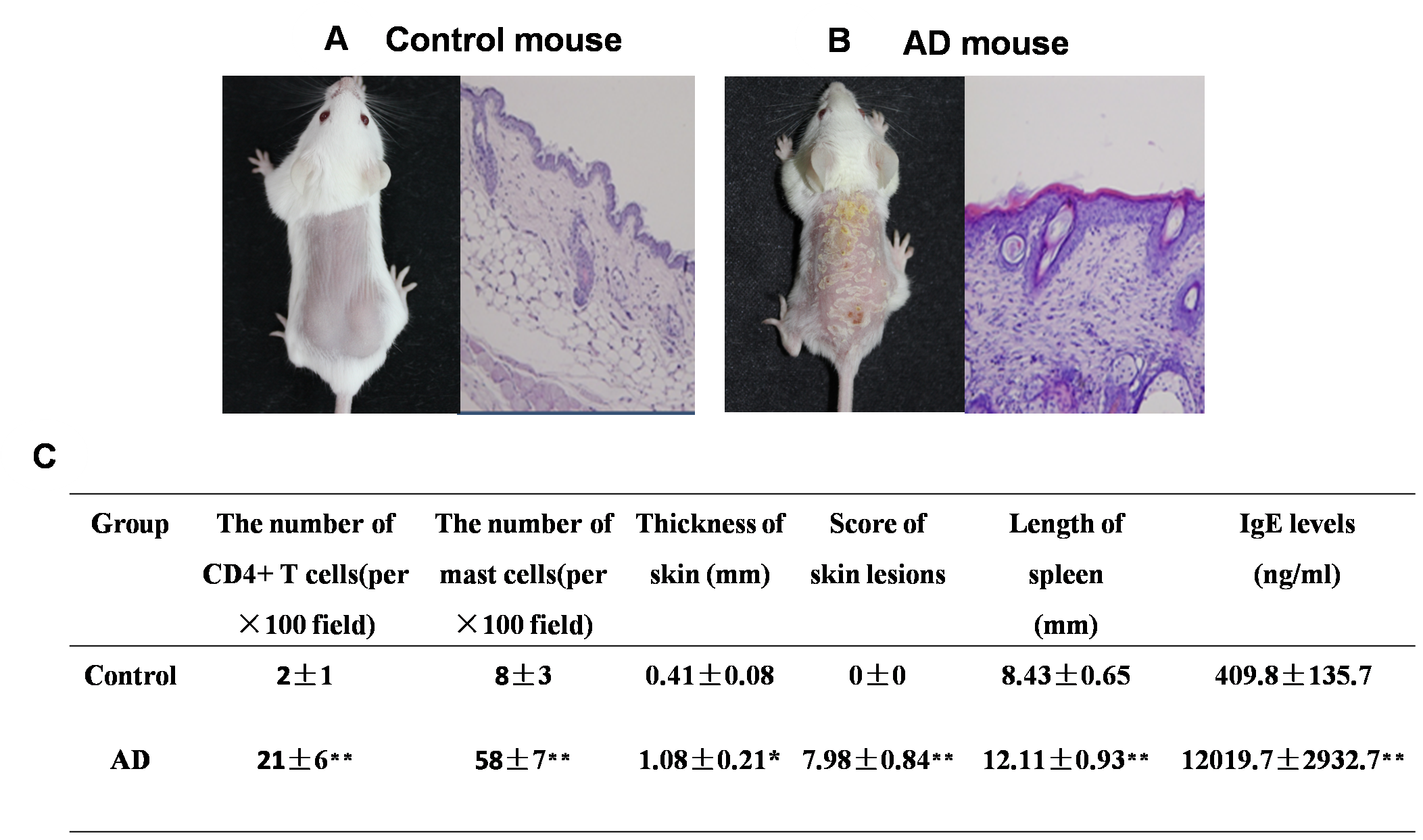

Supplement: S1 File — (Figure A) Skin lesionsand histopathological images of corresponding skin of control mouse; (Figure B) Skin lesionsand histopathological images of corresponding skinofAD mouse. Significant erythema, desquamation, and crusting could be seen on the dorsal skin of model group; (Figure C) Comparison ofthe number of CD4+ T cells (per 100× field), the number of mast cells(per 100 × field), the thickness of skin, the score of skin lesions, the length of the spleen, and the IgE levels between control (n = 10) and AD (n = 10) groups. Data are expressed as mean ± SEM. *P < 0.05, **P < 0.01, AD vs.control. (TIF) [file pone.0132173.s001.tif]
